# Mass Spectrometry Investigation of Some ATP-Binding Cassette (ABC) Proteins

**DOI:** 10.3390/medicina60020200

**Published:** 2024-01-24

**Authors:** Marco Agostini, Pietro Traldi, Mahmoud Hamdan

**Affiliations:** Corso Stati Uniti 4, Istituto di Ricerca Pediatrica Città della Speranza, 35100 Padova, Italy; m.agostini@unipd.it (M.A.);

**Keywords:** mass spectrometry multidrug efflux pumps, (ABC) transporter proteins, multidrug resistance, ion mobility

## Abstract

Drug resistance remains one of the main causes of poor outcome in cancer therapy. It is also becoming evident that drug resistance to both chemotherapy and to antibiotics is driven by more than one mechanism. So far, there are at least eight recognized mechanisms behind such resistance. In this review, we choose to discuss one of these mechanisms, which is known to be partially driven by a class of transmembrane proteins known as ATP-binding cassette (ABC) transporters. In normal tissues, ABC transporters protect the cells from the toxic effects of xenobiotics, whereas in tumor cells, they reduce the intracellular concentrations of anticancer drugs, which ultimately leads to the emergence of multidrug resistance (MDR). A deeper understanding of the structures and the biology of these proteins is central to current efforts to circumvent resistance to both chemotherapy, targeted therapy, and antibiotics. Understanding the biology and the function of these proteins requires detailed structural and conformational information for this class of membrane proteins. For many years, such structural information has been mainly provided by X-ray crystallography and cryo-electron microscopy. More recently, mass spectrometry-based methods assumed an important role in the area of structural and conformational characterization of this class of proteins. The contribution of this technique to structural biology has been enhanced by its combination with liquid chromatography and ion mobility, as well as more refined labelling protocols and the use of more efficient fragmentation methods, which allow the detection and localization of labile post-translational modifications. In this review, we discuss the contribution of mass spectrometry to efforts to characterize some members of the ATP-binding cassette (ABC) proteins and why such a contribution is relevant to efforts to clarify the link between the overexpression of these proteins and the most widespread mechanism of chemoresistance.

## 1. Introduction

Resistance to chemotherapy and molecularly targeted therapy remains one of the major challenges in today’s oncology research. The current scientific literature indicates that resistance to a broad range of anticancer and antimicrobial drugs is related to the level of expression of one or more efflux pump proteins. These are membrane proteins that mediate various functions in their cellular environment, including the transport of noxious substances to the external environment. Membrane proteins, which this group belongs to, comprise about 30% of human proteins [1,2], yet almost 60% of the therapeutic targets belong to this class of proteins [3,4]. Despite their huge potential as therapeutic targets and their wide diffusion in nature, only ~1% of the structures in the Protein Data Bank are of transmembrane proteins. This evident discrepancy is commonly attributed to their physicochemical and biochemical characteristics as well as the additional problems related to their lipid environment, which limit the number of techniques suitable for their structural characterization [5].

Efflux pumps can not only expel a wide range of therapeutic compounds owing to their multi-substrate specificity, but also drive the acquisition of additional resistance mechanisms by lowering intracellular drug concentration and promoting mutation accumulation. The current literature classifies efflux pumps into five structural families, namely the resistance-nodulation-division (RND), the small multidrug resistance (SMR), the multi antimicrobial extrusion (MATE), the major facilitator superfamily (MFS), and the ATP-binding cassette (ABC) superfamilies [6]. It is relevant to point out that the basal level of expression of these proteins varies from one efflux pump to another. This level of expression can be influenced by the presence of compounds or conditions (effectors) [7]. Identification of effectors, which can trigger the expression of the genes that encode efflux pumps, is highly relevant to the understanding of what is known as transient reduction in the susceptibility to antibiotics [8]. Such information is not easily obtained using common susceptibility methods [9].

The ATP-binding cassette (ABC) transporter family of transmembrane proteins is capable of regulating the flux across the plasma membrane of structurally different chemotherapeutic agents. So far, there are 48 known members of this family [10]; however, only three members have been investigated in some detail, with a particular focus on their link to antineoplastic resistance, i.e., multidrug resistance (MDR). These three proteins are multidrug resistance protein 1 (MDR1; also known as P-glycoprotein and ABCB1), MDR-associated protein 1 (MRP1; also known as ABCC1), and breast cancer resistance protein (BCRP; also known as ABCG2). All three proteins have broad, overlapping substrate specificity and promote the elimination of various hydrophobic compounds, including major cancer chemotherapeutics [10]. The first ABC transporter to be identified was P-glycoprotein (P-gp). This membrane-bound glycoprotein is expressed at relatively low levels in most tissues; however, the same protein is found at much higher levels on the surface of epithelial cells with excretory roles, such as those lining the colon and the small intestine [11,12]. (P-gp) overexpression has been associated with chemotherapy failure in many cancers, including kidney, colon, and liver [13].

The insolubility of these proteins in water renders their purification and crystallization in preparation for their analyses highly demanding. Structural characterization of some members of the binding cassette (ABC) transporters is obtained using X-ray crystallography [5], cryo-electron microscopy [14,15], and, more recently, mass spectrometry (MS) [16,17]. In recent years, mass spectrometry (MS) has assumed a significant role in the characterization of membrane proteins. This emerging role can be attributed to a number of reasons, including the availability of MS-compatible detergents able to efficiently solubilize membrane proteins within the investigated samples, maintaining their stability in solution. These detergents have the advantage of being easily removed after the transfer of the precursor ions from solution into the gas phase in the mass spectrometer without significantly impacting on the structure/stoichiometry of the investigated proteins [18]. Additionally, advances in MS instrumentation and supporting bioinformatics have allowed for more sensitivity, higher mass resolution, and improved mass accuracy. Furthermore, the coupling of electrospray ionization (ESI) with ion mobility–mass spectrometry [19,20,21] and the use of isotope labelling (e.g., H/D exchange) [22] facilitated better structural and conformational characterization of these macromolecules. Mass spectrometry is unique in its capabilities, which can be applied to investigate proteins from different angles. This technique can be used to explore an amino acid’s composition, associated post-translational modifications (PTMs), protein–protein interaction, protein assemblies, protein–ligand interaction, and protein conformational changes both in the liquid and in the gas phase.

In this review, we give a brief description of a number of mass spectrometry developments which have enabled this technique to become a major player in the analysis of this class of proteins. Recent examples on the application of this technique and the value of the generated data in the overall attempts to characterize some transporter proteins are discussed. We also discuss how the absence of comprehensive structural/conformational information and the scarcity of computational modelling studies, including molecular docking, could be partly responsible for the failure to discover specific and effective inhibitors to counter the overexpression of transporter proteins. Such computational studies, in particular those based on experimental data, are necessary to extend our knowledge of the functioning mechanism of the investigated proteins and to complement the snapshots acquired from high-resolution 3D structures.

## 2. Discussion

### 2.1. Developments Which Enhanced the Role of MS in Proteins Characterization

The introduction of electrospray ionization (ESI) [23] and matrix-assisted laser desorption/ionization (MALDI) [24] together with the coupling of liquid chromatography (LC) to mass spectrometry (MS) represented a major step towards the use of mass spectrometry to investigate macromolecules. Over the last decade, the application of a number of developments contributed to an enhanced role of MS-based methods in the structural characterization of biomolecules, including membrane proteins. Before discussing some of the main developments we ought to point out the important contribution of improved protein labeling methods. Proper sample preparation, including labeling for MS-based analysis, is a crucial step in any proteomics workflow. In the past decade, relative quantification using various labeling protocols has developed into a key tool for the determination of protein expression and quantification in biological samples. As mentioned in other parts of this review, accurate determination of the level of expression of (ABC) transporters is critical in the evaluation of the link between these levels and multidrug resistance. Over the last two decades a long list of in vitro chemical labeling has been tested. Discussing these protocols in detail is not within the scope of this review; however, indicating some of these methods and associated references may interest some readers. Isotope-coded affinity tags (ICATs) were introduced over 20 years ago [25]. This labeling method has proved to be highly suitable for the quantitative analysis of proteins within complex mixtures. Another method which proved to be useful for the analysis of protein mixtures is known as cleavable ^13^C-isotope-coded affinity tags (cICATs) [26]. Other labeling schemes included dimethyl labeling [27], isotope-coded protein labeling (ICPL) [28], and isobaric labeling [29]. Methods for in vivo labeling use stable isotopes, which can be metabolically incorporated into living systems. The most representative method in this approach is stable isotope labeling by amino acids in cell culture (SILAC) [30].

Two of the main developments which enhanced the role of MS-based methods in structural investigations of macromolecules are discussed below.

#### 2.1.1. Hydrogen–Deuterium Exchange Mass Spectrometry(H/DX-MS)

**Figure 1:**
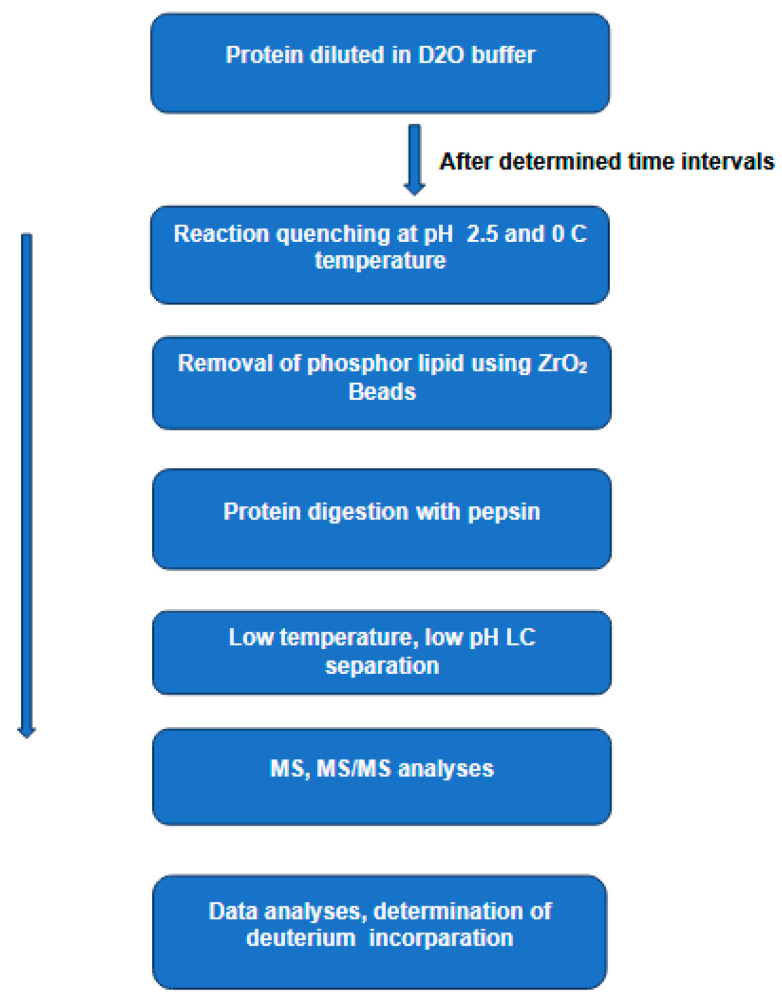
The main steps in hydrogen–deuterium exchange combined with mass spectrometry HDX-MS to study protein conformations in solution. In this technique, a protein is diluted in a deuterated buffer, enabling H/D exchange of labile backbone amides. The process of isotopic exchange is strongly dependent on the protein secondary structure and solvent accessibility. The quenched protein sample is then digested by pepsin and the resulting peptides are separated by LC at low temperature and low pH. The extent of the deuterium incorporation is determined by MS and MS/MS analyses.

In recent years, hydrogen–deuterium exchange combined with mass spectrometry (HDX-MS) [31] has emerged as a key player in studying the conformational dynamics and interaction of proteins in solution [16]. In this technique, a protein is diluted in a deuterated buffer, enabling H/D exchange of labile backbone amides. This process of isotopic exchange is strongly dependent on the protein secondary structure and solvent accessibility. In general, the exchange reaction can be quenched by dropping the pH and the temperature of the solution to 2.5 and 0 °C, respectively, conditions necessary to reduce the D-to-H back exchange. The quenched protein sample is then digested by an acid protease (e.g., pepsin), and the resulting peptides are separated by LC at low temperature and low pH, followed by MS and MS/MS analyses to determine the extent of deuterium incorporation (Figure 1).

The lipid environment of membrane proteins is one of the main problems in HDX-MS analyses. This is because lipids hamper both the digestion process and the subsequent LC separation of the resulting peptides. Present day analyses of these proteins rely on the removal of the phospholipids prior to digestion, and this removal is normally carried out using ZrO_2_ coated beads [32], size exclusion chromatography [33], or trichloroacetic acid (TCA) precipitation [34]. In a recent work [32], the authors described the integration of a chromatographic phospholipid trap column into the experimental arrangement used for online sample delipidation prior to protease digestion of deuterium-labeled protein–lipid assemblies. The authors also used the same arrangement to determine the efficiency of phospholipid capture for both ZrO_2_- and TiO_2_-coated beads. The same study evaluated solution conditions to optimize both online and offline delipidation.

Analyses of proteins containing certain post-translational modifications (PTMs), such as glycosylation and disulfide bonds can be challenging for HDX-MS. The presence of disulfide bonds can be responsible for poor digestion, which leads to poor sequence coverage. Furthermore, the MS/MS spectra generated in the presence of disulfide-linked peptides are not easy to interpret [35,36]. Although at room temperature and neutral pH disulfide bonds can be reduced by a variety of reagents, the low temperature and acidic pH used in HDX quenching narrows down the choice to a single reducing reagent, tris(2-carboxyethyl) phosphine (TCEP) [37]. Still, TCEP has its shortcomings related to the alkaline pH required for optimal disulfide reduction by this reagent. Various works have attempted to find alternative solutions to TCEP reduction. A recent work claimed that electrochemical reduction was demonstrated to be robust and repeatable for online disulfide bond reduction within the HDX-MS procedure [38]. In an earlier work [36], the authors reported that HDX-MS measurements were performed using glycoprotein transferrin. Following pepsin digestion, the resulting peptides were examined by liquid chromatography–tandem mass spectrometry (LC-MS/MS) without prior reduction of the disulfide-linked peptide dimers. Instead, the dissociation of disulfide-linked peptide dimers produced by peptic digestion was carried out using electron capture dissociation (ECD). In other words, the reduction of these bonds was carried out purely in the gas phase rather than in solution. It has to be said that this work is a good example of a proof of concept; however, to consolidate its conclusions, further examples involving a larger number of different proteins are necessary.

Glycosylation is another PTM, which requires more demanding HDX-MS and MS/MS analyses. This is because a single glycosylation site can be occupied by multiple, heterogeneous glycated structures, which inevitably results in different glycoforms of the same proteoform. This effect causes glycopeptide signal distribution and inevitable signal reduction in the individual structures [39]. Collision-induced dissociation of glycopeptides is also problematic because of the preferential cleavage of glycosidic bonds in carbohydrate moieties [40,41]. The same authors pointed out that the use of ETD instead of CID did not improve the signal [42].

In recent years there has been an increasing use of HDX-MS in the investigation of integral membrane proteins [43]. As mentioned earlier, this class of proteins is known to perform a range of diverse functions, and their dysfunctions are frequently linked to numerous diseases, which explains the huge interest of the pharmaceutical industry in these proteins as promising therapeutic targets. Over the last decade, HDX-MS has developed into a versatile tool for probing the protein dynamics of integral membrane proteins in solution. Earlier investigations included detergents’ extraction of the investigated proteins from their native environment, a step which is likely to interfere with the native structure. One of the approaches to simulate the native environment is the development of membrane mimetics [44]. Over the last two decades various research efforts managed to expand the diversity of membrane mimetics and to use more lipid bilayer-like structures, which, together with the incorporated proteins, have high solubility and stability [45]. The combination of H/DX with mass spectrometry was used to investigate the mechanism of inhibition by targeting the adaptor protein, AcrA. This protein is part of the resistance-nodulation-cell-division (RND) efflux pumps family, which is strongly linked to resistance to a wide range of antibiotics contributing to multidrug resistance (MDR) [46,47,48]. This membrane protein is part of a tripartite complex, AcrAB-TOLC, which consists of the AcrB transporter, embedded in the inner membrane, the AcrA adapter located in the periplasm, and the channel protein TOLC responsible for the transport of substrates towards the extracellular environment. In addition to conferring resistance to many classes of antibiotics, this complex plays a role in the pathogenesis and virulence of several bacterial pathogens. The same complex is a target for inhibition to interfere with resistance development and restore antibiotic efficacy. MS analyses of this complex furnished molecular insights into multidrug adaptor protein function which could be useful for the development of new antimicrobial therapeutics [49].

In the last few years, most investigations of protein conformational changes by HDX-MS have been conducted in vitro. This is because the experimental conditions for such analyses can be optimized to minimize back exchange prior to MS analyses. Performing similar measurements in vivo demands more stringent experimental conditions to prevent excessive back exchange of deuterium. In vivo measurements are necessary to gain a better insight into the dynamics and functions of the investigated proteins in their natural environment. Isolating proteins from their membranes using detergents into membrane mimetic systems may vary their functions and is not representative of their state *in vivo*. The current literature shows emerging attempts to perform HDX-MS in vivo. In a recent article, a research group developed a protocol for in vivo HDX-MS using as a model BtuB, an *E-coli* outer membrane protein, known to be involved in vitamin B12 transportation [50]. The main steps in this protocol were as follows: living *E. coli* cells overexpressing BtuB protein were diluted in D_2_O buffer supplemented with a carbon source to prevent starvation during analysis and were then examined by HDX-MS. The reaction was then quenched by dropping the buffer pH to 2.5 and flash freezing the cells with liquid nitrogen; the latter step also acted to lyse the cells via cryogenic disruption. Proteins of interest were then isolated by ultracentrifugation at pH 2.5 and 2 °C. This was followed by in-solution proteolytic digestion, the addition of ZrO_2_ for delipidation, and the removal of peptides via centrifugal spin filtration. Peptides were then subjected to LC-MS/MS analyses. The authors reported that in vivo and in vitro HDX-MS experiments provided high levels of BtuB sequence coverage (93% and 98%, respectively). An earlier example on the use of DHX-MS for in vivo protein analyses was reported [34]. The authors investigated outer membrane vesicles naturally released by *Escherichia coli*. A protocol was developed which allowed the removal of most of the lipid content while limiting the back exchange.

Future in vivo analyses using HDX-MS will no doubt furnish much needed structural and conformational information of integral membrane proteins. Such information will shed more light on the biology of these proteins, which in turn will help in the identification of specific therapeutic targets within such a large family of proteins.

#### 2.1.2. Ion Mobility–Mass Spectrometry

Over the last 20 years the use of ion mobility in combination with mass spectrometry (IM-MS) to investigate macromolecules has furnished valuable information and deeper insight into the field of structural biology. This technique is considered an additional dimension to analyses performed by the LC-MS and MS/MS platform. The combination of ESI-MS/MS and IM-MS is a powerful tool for the resolution and identification of complex protein digests. Furthermore, such analytical platforms can provide much needed information on the confirmational states of a wide range of proteins. Before discussing the role of IM-MS in macromolecular analyses, there are two observations to consider. First, IM separates gas-phase macromolecular ions according to their mass, charge, size, and shape. While mass and charge can be determined by mass spectrometry (MS), it is the addition of ion mobility that enables the separation of isomeric and isobaric ions and the direct elucidation of conformation. Second, this technique has been around for over 50 years [51], and in the early years of its introduction it was mainly used to study gas-phase ion-molecule reactions to determine their rate constants [52]. The combination of this technique with commercially available mass spectrometers to investigate biomolecules appeared over a decade ago [53].

Under the general title “ion mobility”, there are different hardware configurations, including drift tube ion mobility, cyclic ion mobility, trapped ion mobility spectrometry, and differential ion mobility and field asymmetric waveform ion mobility spectrometry. All these ion mobility variants operate on the same basic principle, i.e., the separation of ions in a buffer gas (normally helium or nitrogen) under the influence of a relatively weak electric field. Under such conditions, the drift time of an ion is dependent on its mass, charge, size, and shape. Large, more structured ions experience more collisions with the buffer gas and, therefore, have longer drift times. The drift time of a given ion can be converted into a parameter known as the collision cross-section (CCS) [54], a parameter, which reflects the extent of interaction (collisions) between the drifting ion and the buffer gas. Experimentally determined CCS values can also be compared with those generated by computational modelling methods, such as structure prediction from sequence. Various studies have demonstrated that CCS is less prone to variations in the experimental conditions applied in different labs and using different instruments, which renders this parameter an ideal candidate for the standardization of results derived from different sources, a characteristic of high value in biomedical research [55,56]. Recently, ion mobility–mass spectrometry (IM–MS) has been shown to be a highly promising technique for the identification of untargeted metabolites present in biological samples. It is interesting to note that the separation and identification of such metabolites is mainly based on differences in their collision cross-sections (CCSs) [57,58].

**Figure 2:**
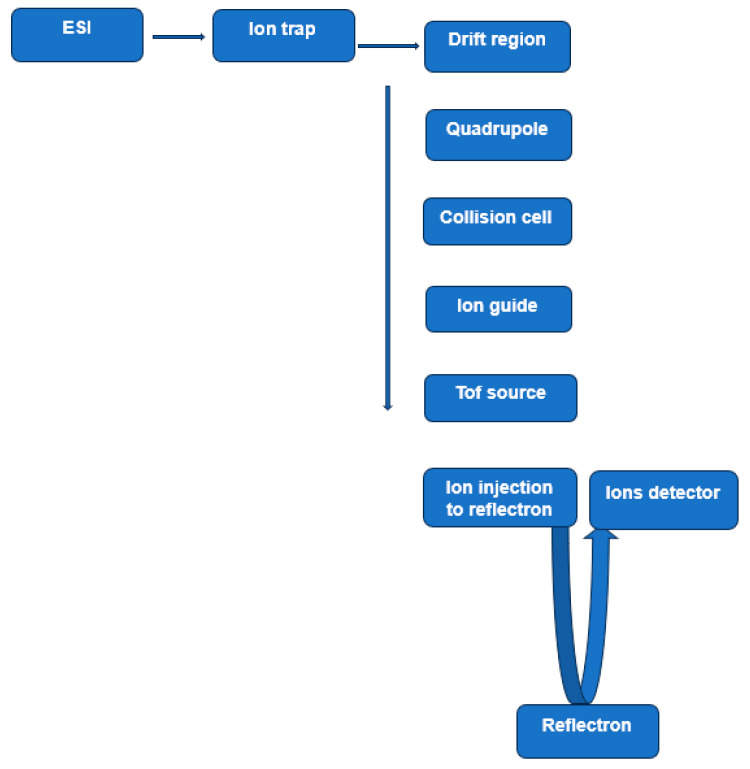
Ion trap/quadrupole/time-of-flight (Q-TOF) mass spectrometer. This experimental arrangement includes an electrospray ionization source (ESI), a drift tube to perform ion mobility, a quadrupole mass filter, a collision cell, and a reflectron TOF. Based on Figure 2 [59].

A hybrid IM-MS system consisting of an ion trap, drift tube, quadrupole, and time-of-flight is given in Figure 2 [59]. The general sequence of the analysis steps in such a configuration is as follows. Ions formed by ESI are extracted into the high vacuum region and accumulated in the first quadrupole ion trap. The accumulated ions are then injected into the drift region by applying short ion pulses. The injected ions move in a buffer gas driven by a weak electric field. In this region, ions are separated according to their charge, mass, size, and shape. Ions exiting the drift region are focused into a quadrupole mass filter, which can be tuned to acquire the desired ion transmission. Ions exiting the quadrupole are decelerated to the desired energy and focused into a collision cell for CID analyses. Remaining precursor ions and fragment ions exit the collision cell to be focused into the source region of a reflection geometry time-of-flight (TOF) mass spectrometer, where the ions are detected.

Recent research demonstrated that IM-MS is capable of providing structural information, which for various reasons could not be provided by high-resolution techniques, such as X-ray crystallography and cryo-electron microscopy (cryo-EM). One of the interesting characteristics of IM-MS is that the transition of protein/peptide ions from the solution to gas phase does not seem to influence the secondary structure, compactness, or the quaternary structure of the investigated proteins [60,61]. In a relatively recent work [62], the authors used experimental ion mobility data in a computational modelling approach to predict protein structure. The use of experimentally generated data in computational modelling methods, such as structure prediction from sequence or protein–protein docking can provide insight into structural details at the atomic level, details which cannot be obtained directly from the experimental datasets. For example, molecular dynamics and molecular docking were used for a more detailed characterization of the ABCG2 homodimer [63]. The role of key residues and motifs in the structural stability of this dimer were comprehensively studied and were found to be in good agreement with existing experimental data. In a more recent review [64], the authors described in some detail the typical features of an IM-MS experiment from the preparation of samples, the formation of ions, and their separation in different mobility and mass spectrometers. The same review described the interpretation of ion mobility data in terms of protein conformation and how the experimental data can be compared with data from other sources with the use of computational tools.

#### 2.1.3. More Efficient Activation Methods of Macromolecules

For over 40 years, tandem mass spectrometry (MS/MS) has been performed by accelerating a mass selected precursor ion into a collision cell containing a neutral gas (Ar, N2) maintained at pressure approximately 10^−4^ to 10^−2^ torr and a collision energy of 10–100 eV. Such collision conditions are commonly used in triple quadrupoles [65], ion traps, and hybrid time-of-flight (Q-TOF). Over the last twenty years, softer and more efficient fragmentation methods have been introduced as alternatives or as complementary techniques to collision-induced dissociation (CID). These methods include electron transfer dissociation (ETD), electron capture dissociation (ECD), and photodissociation. Over the last 20 years, these methods have been demonstrated to be highly suitable for the investigation of peptides/protein sequencing, particularly those with labile post-translational modifications (PTMs). A number of studies have demonstrated that the use of these methods alongside CID improves the sequence coverage, and, due to lower energy compared to CID, the same methods tend to preserve most labile PTMs, facilitating more reliable localization of the site of modification.

#### 2.1.4. Electron Capture Dissociation (ECD)

Over the last twenty years, electron capture (ECD) and electron transfer dissociation (ETD) have emerged as two of the most useful methods for peptide/protein fragmentation. The diffused use of both fragmentation methods coincided with unprecedented advances in mass spectrometry and liquid chromatography. In the ECD method, low-energy (less than 1 eV) electrons are captured by multiply charged protein/peptide positive ions generated by ESI [66]. This exothermic reaction induces dissociation through a H atom transfer to the backbone carbonyl group, resulting in a relatively soft cleavage of the C_α_–N bond in a peptide backbone. Such low-energy interaction allows for much better detection and localization of labile PTMs as well as more sequencing information compared to classical CID. The predominant bonds cleaved in such a reaction are the backbone N–Cα, yielding *c* and *z* ions, with the exception of N-terminal proline cleavage, due to the proline’s cyclic nature [67,68]

Since its introduction, the application of ECD has been limited to Fourier transform ion cyclotron resonance instruments (FT-ICR) [69]. The main reason for such a limitation is that efficient ECD requires a dense population of thermal electrons to interact with the precursor ions for a given time window. Creating such conditions in instruments, which use radio frequency to trap the precursor ions (e.g., ion traps and hybrid Q-TOF), proved to be technically challenging. In the last ten years, however, various attempts have been made to use ECD in other types of instruments, including hybrid Q-TOF and ion traps [70]. An electro magnetostatic cell, which uses a magnetic field to confine low-energy electrons in a flight path of the precursor ion, facilitating ECD fragmentation without the need for long reaction times or ultrahigh vacuum has been reported [71,72]. An orbitrap instrument was modified to house an electro-magnetostatic cell to perform ECD analyses. It is relevant to note that both ECD and ETD deliver richer sequencing information when the precursor ion has high charge states.

#### 2.1.5. Electron Transfer Dissociation (ETD)

Electron transfer dissociation (ETD) [73] is the direct result of interaction between multiply charged positive ions generated by electrospray ionization and a negatively charged radical reagent. The chemical properties of the radical reagent and the charge density of the precursor ion are the two parameters which influence the reaction. The two reaction pathways are deprotonation of the multiply charged ion and electron transfer from the radical reagent to the multiply charged ion (peptide or protein). The reactive radical anions are generated from polycyclic aromatic hydrocarbon molecules, such as anthracene or fluoranthene. The latter is considered as one of the more favorable ETD reagents. Electron transfer from the negatively charged reagent ions to multiply charged positive precursor ions induces dissociation of C_α_–N bonds in a similar fashion to that observed in ECD. Both ETD and ECD are low-energy reactions, which explains their capability to detect and localize labile post-translational modifications, which are difficult to detect in collision-induced dissociation. That said, obtaining rich sequencing information by electron-based methods is closely related to the charge density of the precursor ion. Such influence manifests itself clearly in low charge density ions, where backbone cleavage takes place, but the resulting fragment ions are held together by non-covalent interactions present in the more compact structures, a process known as non-dissociative electron transfer [74]. One way to overcome such shortcoming is to chemically modify the investigated peptides [75,76] or to conduct combined ETD and CID, where the first method is used for high charge states, while the second is used for lower mass lower charge states [77,78]. Another relevant observation regarding peptide sequencing using ETD concerns the link between the length of the peptide and the sequence coverage. At room temperature, very high sequence coverage (~100%) was observed for small peptides (≤7 amino acids). For medium-sized peptides composed of 8−11 amino acids, the average sequence coverage was 46%. Larger peptides with 14 or more amino acids yielded an average sequence coverage of 23% [74]. The same study showed that higher temperature ETD provided increased sequence coverage over room-temperature experiments for peptides of greater than 7 residues.

For in-depth discussion of radical-mediated gas-phase chemistry and the application of ETD and related techniques in sequencing studies, the readers are referred to more comprehensive reviews [69,79,80].

#### 2.1.6. Photodissociation Methods

Currently there are two main photon-based methods for the activation and subsequent fragmentation of macromolecular ions. The first method uses infrared multiphoton dissociation (IRMPD), which was first demonstrated almost 50 years ago [81]. The authors used the wavelength 10.6 μm from a tunable continuous wave CO_2_ laser to irradiate a proton-bound dimer of di-Et ether [(C2H5)2O]2H^+^. The measurements were conducted in Fourier transform ion cyclotron resonance (FTICR). For many years the use of IRMPD remained limited to fundamental studies of small molecules; however, more recently, it is increasingly being applied to the analyses of macromolecules. This increase in the use of IRMPD can be attributed to advances in the characteristics of IR lasers, particularly the IR optics. The extension of this photodissociation method to other mass spectrometers, including linear and quadrupole ion traps, orbitraps, and hybrid TOFs is another reason for the increasing use of this method in peptide/protein fragmentation This activation method combined with tandem mass spectrometry (MS/MS) can provide fairly precise information on the type and sites of various PTMs of individual amino acids as well as on amino acids within peptides and proteins. Some of the modifications examined by this method include phosphorylation, sulfation, acetylation, methylation, glycosylation, and disulfide bond formation [82,83]. The photodissociation of the precursor molecular ions containing one or more of these PTMs is induced by the presence of one or more reactant group(s) (e.g., -OH, -C-H, -S-H, -S-CH_3_) responsible for the absorption of the irradiating photons. In this method, interaction between IR photons and multiply charged precursor ions induces multiple photon absorption and progressive increase in the precursor ion internal energy, resulting in various bonds cleavage. The number of photons necessary to reach a dissociative vibrational level(s) of the precursor ion depends on the energy of the photons and the duration of the irradiating pulse. Such fragmentation generates a series of b- and y-ions similar to those generated in the CID method. This observation is rather expected since both mechanisms of fragmentation proceed through dissociative vibrational excitation of the precursor ion.

The second method uses ultraviolet photodissociation (UVPD) at different wavelengths. For instance, 193 nm was first applied in the 1980s [84,85]; however, such an application was limited to specific peptides performed on Fourier transform ion cyclotron resonance (FTICR) mass spectrometers. Over the last ten years and due to advances in UV lasers and in MS instrumentation, the technique has witnessed a resurgence, represented in its use in a wide range of instruments, including TOF [86], linear ion traps [87], and more recently in the orbitrap [88]. UVPD mainly uses two wave lengths, 157 and 193 nm; however, the wavelength 213 nm has been used to sequence intact proteoforms in top-down proteomics [89]. In UVPD, due to the absorption of a single photon having 5–7 eV energy by the precursor ion, fragmentation proceeds either through electronic dissociative excitation or through the interconversion of the electronic excitation to vibrational excitation, thus leading to bond rapture, which is the major fragmentation pathway [90,91]. Various works have also demonstrated that fast UVPD generates a, b, c, x, y, and z sequence ions, in addition to v and w side-chain loss ions [86] The same studies indicated that UVPD at 213 nm not only offered a higher sequence coverage compared to the well-established high-energy collision dissociation (HCD) method but also offered a better chance to detect and localize PTMs.

As mentioned above, in present-day MS/MS analyses, there are different fragmentation methods to investigate peptide/protein fragmentation. One of the obvious questions is as follows: why do we need multiple fragmentation methods to investigate protein digests? This question has different answers. First, none of the existing activation methods can provide a comprehensive sequencing information on the various components and associated PTMs within a complex protein digest on their own. Second, it is well recognized that protein mixtures tend to contain various labile PTMs, a situation which dictates the choice of an activation method suitable for the detection and localization of such modifications. The need to use multiple activation methods is reflected in what can be described as a new trend in MS instruments. Such instruments are equipped with various components to allow different modes of precursor ion activation. In a recent work, a segmented linear ion trap to perform multidimensional, multiple-stage tandem mass spectrometry was described [92]. Precursor ion activation included injection of reagent ions, radical neutral species, photons, and electrons. Another recent work described an omnitrap–orbitrap platform equipped with infrared multiphoton dissociation, ultraviolet photodissociation, and electron capture dissociation for the analysis of peptides and proteins [88]. This new trend in commercial MS instrumentation is good news for research labs and industries, who can afford to purchase such systems. For small labs there is always the option of home-made systems.

## 3. Analysis of Some ATP-Binding Cassette (ABC) Transporters

The introduction of two soft ionization techniques, namely electrospray ionization (ESI) [23] and matrix-assisted laser desorption/ionization (MALDI) [24], was a major step towards a diffused use of mass spectrometry (MS) in biological and biochemical worlds. Over the last two decades, advances in MS instrumentation together with more refined labelling protocols, the combination of ion mobility with MS, and more frequent use of electron-based and photon-based fragmentation methods paved the way for what is known today as structural mass spectrometry. With these innovative developments, mass spectrometry can be applied for the structural characterization of a wide range of macromolecules and in some cases their respective assemblies. It is relevant to point out that continuous progress in sample preparations, including the use of a new class of detergents, extended the application of MS to membrane proteins, which are not water soluble. In today’s world, MS analyses provide valuable information on protein structures, conformations, noncovalent protein–protein assemblies, protein–ligand complexes, and quantitative protein expression in biological samples derived from patients suffering from various diseases.

### 3.1. Monitoring the Conformation of P-glycoprotein

**Figure 3:**
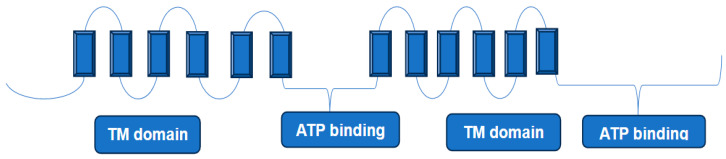
Simple representation of P-glycoprotein structure. X-ray structure shows two transmembrane domains (TM domain) and two nucleotide-binding domains (not shown here) and adenosine triphosphate (ATP) (based on Figure 1 in [103]).

P-glycoprotein (also known as ABCB1 and MDR1) is the first identified mammalian ABC transporter protein, discovered almost half a century ago [93]. It has a ~170 kDa molecular weight and is comprised of two homologous halves, each containing a nucleotide-binding domain (NBD) and a transmembrane domain (TMD) [94]. The transmembrane domain contains a hydrophobic cavity, accessed by portals in the membrane, that binds cytotoxic compounds as well as lipids. This protein is expressed in multiple key organs, such as the small intestine, blood–brain barrier, kidney, and liver. Therefore, P-glycoprotein mediated drug–drug interactions can occur in various organs and in tissues. The overexpression of P-glycoprotein has been linked to chemotherapy failure in various cancers, including kidney, colon, and liver. A hallmark characteristic of this transporter protein is its ability to bind and transport a wide range of structurally different molecules in the molecular mass range, 100 to 4000 Da, a range which covers most if not all anticancer and antimicrobial drugs currently in use [95]. The transportation of these molecules across the membrane was found to coincide with changes in the size and shape of a large multi-specific drug-binding pocket [94]. Although its physiological function(s) are yet to be fully understood, the well-recognized role of this protein in mediating multidrug resistance in many types of cancers has made it an attractive therapeutic target [96]. The structure of P-glycoprotein has been extensively studied, and consists of two homologous segments connected by a flexible linker on a single polypeptide chain (Figure 3). Existing studies indicate that during the transport cycle, such a structure alternates between two distinct conformational states, an inward-facing conformation capable of binding intracellular transport substrates, and an outward-facing conformation oriented to eject substrates across the membrane [97] (inward/outward refer to the opening of the drug-binding pocket relative to the cell). Molecular dynamics simulations and X-ray structural studies also suggested that in addition to the large amplitude motions associated with switching between inward facing and outward facing conformation states, some regions undergo local fluctuations in their secondary structure, including hinge regions in the transmembrane helices [94,98,99].

In recent years it has been demonstrated that hydrogen–deuterium exchange mass spectrometry (HDX-MS) is ideal for studying protein dynamics as they occur in solution. Proteins are known to assume a wide range of conformations in solution, which means that every amide proton will eventually exchange with deuterium. Measuring this rate of exchange as a function of time would result in a highly informative picture of the regional dynamics. Information provided by this technique are fundamental for the understanding of the functioning mechanism of ABC exporters, including P-gp, and complement the snapshots of the catalytic cycle provided by high-resolution 3D structures.

In a recent article, HDX-MS was used to investigate the conformational states as well as the dynamics and mechanism of transportation of P-glycoprotein. The authors detected three distinct conformational states and obtained information on transporter dynamics with a sequence coverage over 85% [100]. This high sequence coverage and the use of bio-layer interferometry to measure nucleotide affinity furnished further information on the states of conformation, dynamics, and mechanism of transportation by this protein. According to these authors, the most surprising result was the differences in the deuterium uptake of the extracellular loops (ECLs) between the apo, pre-hydrolytic, and outward-facing states. In the pre-hydrolytic state, the dynamics in this region decreased, while the same region increased in terms of dynamics in the outward-facing state when compared to apo conformation. This observation suggests a mechanism which prevents the transporter from behaving as a channel during the intermediate transition between the inward-facing and outward-facing states.

**Figure 4:**
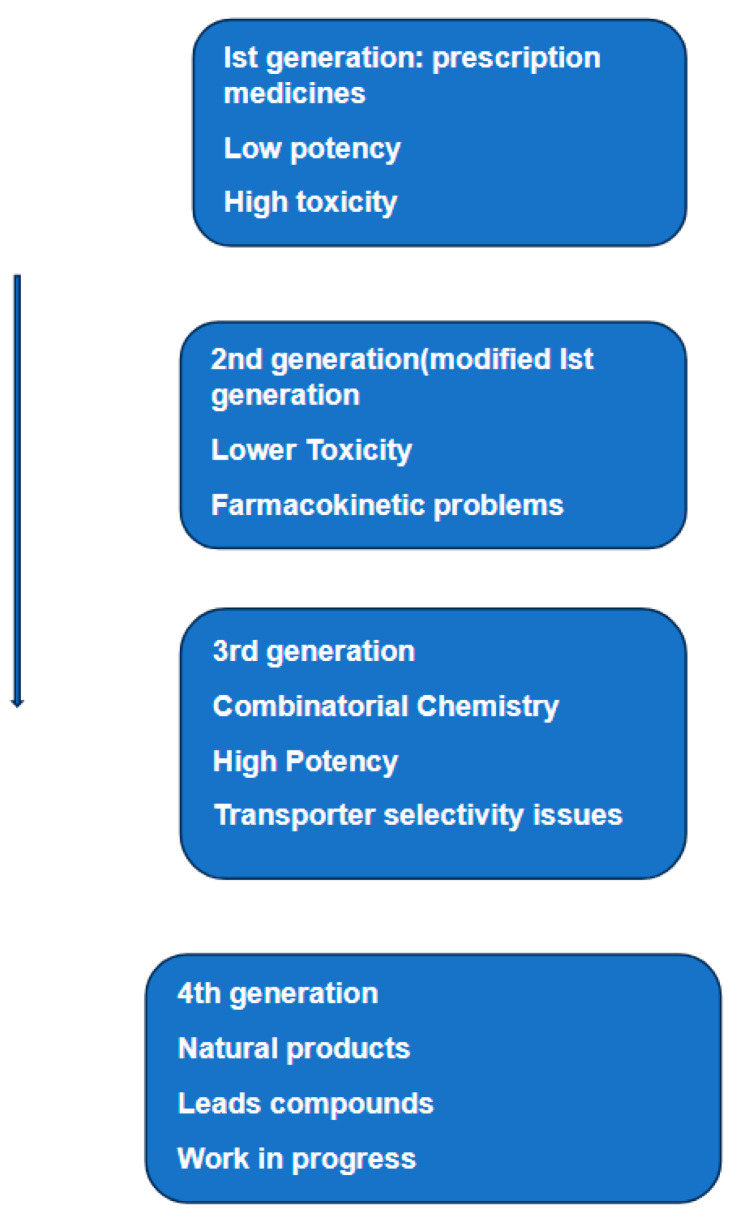
Schematic representation of the various phases of research and development of inhibitors of P-glycoprotein. The same diagram lists the weaknesses of each phase and attempts to correct them. The text related to this diagram concludes that despite 40 years of research, we are still looking for specific and effective inhibitors of P-glycoprotein. Based on Figure 18.1 in [101].

Since its discovery almost 50 years ago, P-glycoprotein has been structurally characterized by X-ray, cryo-electron spectroscopy, and, more recently, by HDX-MS. This structural information, together with data furnished by analyses of clinical samples provided by patients undergoing chemotherapy and molecularly targeted therapy, gives a clearer picture of the role of this protein in MDR. The initial enthusiasm that such new information could lead to the discovery of P-glycoprotein inhibitors was quickly dampened. The reasons for such a disappointing outcome were given by Crowly et al. [101]. The authors divided the development of such inhibitors into four generations (Figure 4). This figure suggests that the failure of these inhibitors to restore sensitivity to chemotherapy reside in their poor selectivity, low potency, inherent toxicity, and/or adverse pharmacokinetic interactions with anticancer drugs. The failure to develop efficient and specific inhibitors of P-gp reflects the multifactorial nature of drug resistance. Data accumulated over sixty years have identified at least eight mechanisms implicated in MDR [102]. Currently, we do not have sufficient information on how these mechanisms interact functionally and under what conditions some of them work synergically. Taking P-glycoprotein as a representative example the following observations can be made: first, the ATP-binding cassette (ABC) transporter family, including P-gp, have broad, overlapping substrate specificity and promote the elimination of various hydrophobic compounds, including most cancer chemotherapeutics in use today. This protein can not only expel a wide range of therapeutic compounds owing to its multi-substrate specificity but can also drive the acquisition of additional resistance mechanisms by lowering intracellular drug concentration and promoting mutation accumulation. Second, the expression of P-gp is known to be influenced or even induced by compounds or conditions known as effectors. Identification of effectors, which can trigger the expression of the gene(s) that encode efflux pumps, is highly relevant to understand what is called a transient reduction in the susceptibility to anticancer drugs. Third, as mentioned above one of the most widespread mechanisms of resistance is the enhanced expression of efflux pumps, such as ABCB1 (P-glycoprotein), ABCC1 (MRP1) and ABCG2 (BCRP) [103,104]. We also know that the mechanism of resistance is a generic one, owing to the ability of efflux pumps to transport a wide range of different molecular structures [105]. Given the differences in the structure between the three proteins [63], would an inhibitor designed specifically to inhibit P-gp work for the other two members? A partial answer to this question was given by Nanayakkara et al. [106]. The authors tested several compounds that inhibit P-gp by targeting its NBD. According to the authors, these compounds showed successful MDR reversal when tested on drug-resistant prostate cancer cell lines. The same study reported the inhibition of P-gp but not the closely related ABCG2 transporter.

Ion mobility coupled to nanoflow electrospray mass spectrometry was used to probe complex formation between the intact P-glycoprotein and small molecules in detergents. The authors reported that the gas-phase ions of the detected complex were free of detergent yet retained drug molecules as well as charged or zwitterionic lipids. One of the results of this study is the detection of a complex between the intact P-gp and two molecules of cyclosporin A, a cyclic peptide inhibitor used to restore the sensitivity of cancer cells to chemotherapeutic agents. Real-time monitoring showed binding of a variety of different lipids, with complexation preference for negatively charged over zwitterionic double-chain lipids. Binding was also monitored for bulkier four-chain cardiolipins where the negative charge promotes binding, with shorter hydrocarbon chains binding preferentially over their longer counterparts. Based on these experimental data, the authors used molecular docking to assemble models of the inward conformation of P-gp bound to cyclosporin A and cardiolipins. The authors concluded that nucleotide and substrate binding are able to perturb the equilibrium between two conformational states, increasing the population of a minor conformation. In essence, this study highlighted the ability to probe ligand binding to membrane proteins in the gas phase and to directly observe allosteric effects.

### 3.2. Breast Cancer Resistance Protein (ABCG2)

The human ATP-binding cassette transporter ABCG2, also known as breast cancer resistance protein (BCRP), is a key player in anticancer resistance and in physiological detoxification across tissue barriers [107]. Despite numerous investigations, the molecular mechanism of substrate transport by this protein remains to be fully clarified. The activities of this protein are known to affect the pharmacokinetics of commonly used drugs as well as interfering in the delivery of various therapeutics into tumor cells, thus contributing to multidrug resistance [108]. Unlike most of the other ABC transporters, which usually have two nucleotide-binding domains and two transmembrane domains, ABCG2 consists of only one nucleotide-binding domain followed by one transmembrane domain. Thus, ABCG2 has been thought to be a half-transporter that may function as a homodimer (molecular weight approximately 144 kDa).

The first high-resolution structure of human ABCG2 determined by cryo-electron microscopy was reported over five years ago [109]. The structure shows two cholesterol molecules bound in the multidrug-binding pocket that is located in a central, hydrophobic, inward-facing translocation pathway between the transmembrane domains. Within the same year, another research group used the X-ray crystal structure of ABCG5/G8 to generate a model of ABCG2 [109]. To validate structural and mechanistic predictions of their model, the authors used extensive molecular–genetic analyses. The ABCG2 structure contains two apparent cavities. The architecture of the central cavity includes the intracellular loop1, the elbow helix, and residues facing the cavity from transmembrane helices and the NBD dimer. The central cavity is part of the transmission interface, which is essential for ABCG2 drug transport. The smaller upper cavity is part of what is called the extracellular polar roof. These two cavities were found to be separated by two leucine residues, facing their equivalent residues in the core of the symmetric ABCG2 dimer [108,109,110,111]. A number of reports maintain that such structural feature indicates that the ABCG2 transport cycle may also engage a gating mechanism that controls substrate movement from the central to the upper cavity along the substrate translocation pathway [112,113]. Detailed characterization of the ABCG2 homodimer was obtained using molecular dynamics simulations and molecular docking studies [63]. In their study, the role of important residues and motifs in the structural stability of the transporter was fully studied and was found to be in good agreement with the existing experimental data. Furthermore, structural motifs potentially involved in signal transmission were identified, along with two symmetrical drug-binding sites.

The link between ABCG2 and resistance to anticancer drugs has been discussed in a number of studies conducted on cancer cell lines. In one of these studies from over 25 years ago, overexpression of ABCG2 in model cancer cell lines (MCF-7 breast cancer cells) was shown to cause resistance to a number of anticancer drugs, including doxorubicin and daunorubicin [114].

Over the last ten years, a new class of targeted anticancer drugs has emerged. One of these drugs, sonidegib (trade name odomzo), was approved by the FDA in 2015 for the treatment of adults with advanced basal cell carcinoma. This drug was tested as an inhibitor of the transport functions of both ABCB1 and ABCG2 [115]. The authors reported that in accumulation studies, the transport functions of both proteins were effectively inhibited by sonidegib. Within the same study, drug combination assays showed sonidegib synergistically enhanced the cytotoxicity of daunorubicin and mitoxantrone in ABCB1- and ABCG2-overexpressing cells, respectively. A similar effect was also observed in tumor cultures derived from patients suffering from non-small cell lung cancer (NSCLC). One of the interesting conclusions in the same study is that the anticancer effects of sonidegib were not hampered by the expression of the ABC transporters associated with MDR.

The link between ABCG2 and multidrug resistance was investigated in vitro using lung and colon cancer cell lines [116]. The authors conducted various experiments to determine the role of MK-2206 in the attenuation of multidrug resistance in cancer cells overexpressing p-glycoprotein and ABCG2 transporters. The compound MK-2206, an inhibitor of the protein kinases AKT1/2/3, is undergoing evaluation in multiple clinical trials for the treatment of various forms of cancer. Following the incubation of cells with MK-2206, the expression of ABCG2 was measured using Western blotting, while immunofluorescence assays were used to determine the localization of the same protein on the cancer cells membranes. The efficacy of MK-2206 as an attenuator of drug resistance was evaluated in combination with anticancer compounds used as substrates of either G-glycoprotein or ABCG2. This in vitro study concluded that MK-2206 significantly increased the efficacy of the anticancer drugs mitoxantrone, SN-38, and topotecan in MDR lung cancer cells overexpressing ABCG2 transporter. The same drug, however, did not result in a significant change in the expression or in the cellular location of the same transporter. Within the same study, docking simulations indicated that MK-2206 binds in the substrate binding pocket of the ABCG2 transporter.

It is interesting to note that P-glycoprotein has been extensively investigated by HDX-MS to elucidate its dynamics and its conformational states in solution. However, the authors of this review are not aware of similar analyses regarding the closely related ABC transporter, ABCG2/BCRP, and we do not have a reasonable explanation for the absence of such relevant analyses. A tentative explanation for such absence might be related to the oligomeric structure, which contains a number of S-S bridges, rendering solubility and subsequent HDX-MS analyses rather challenging. The oligomeric structure of this protein is partially supported by an earlier study, in which a multistep procedure was used to investigate the oligomeric characteristics of human ABCG2 [117]. The authors used an experimental procedure consisting of the non-denaturing detergent perfluorooctanoic acid and Triton X-100 in combination with gel filtration, sucrose density gradient sedimentation, and gel electrophoresis. These analyses revealed that human ABCG2 exists mainly as a tetramer, with a possibility of a higher form of oligomerization. Monomeric and dimeric ABCG2 did not appear to be the minor form of the protein.

## 4. Commenting on Inhibitors of P-glycoprotein

Four generations of potential inhibitors of P-Glycoprotein have been developed and tested over a period of 40 years [104]. During this period, many potential inhibitors were extensively investigated, but none of them obtained the approval of either the FDA (Food and Drug Administration) or the EMA (European Medicines Agency). Most experts in the field argue that the failure of these inhibitors to restore sensitivity to chemotherapy reside in their poor selectivity, low potency, inherent toxicity, and/or adverse pharmacokinetic interaction with anticancer drugs. However, it has to be said that despite the poor results of years of research, a number of studies during the same period indicated that under specific conditions the combination of potential inhibitors of MDR1 and ABCG2 with certain chemotherapeutics resulted in increased drug accumulation and drug resistance reversal. For example, a recent study [118] reported that the inhibition of MDR1 or ABCG2 enabled doxorubicin to eliminate liver cancer stem cells. Doxorubicin is the most frequently used chemotherapeutic agent in the treatment of hepatocellular carcinoma (HCC). However, the efficacy of this agent is found to be limited by the chemoresistance [119]. In other words, this therapy was found to cure the bulk of the tumor but failed to eliminate the stem cell population, which leads to the continuous progression of the disease. It has to be emphasized that these results were mainly based on in vitro investigations, except for a few randomized trials which have also shown benefits with the use of such inhibitors combined with chemotherapy. The failure of over 40 years of research to produce specific and effective inhibitors of P-gp cannot be attributed to a single cause; on the contrary, there must be many factors, some of which are briefly considered below.

**- *In vitro* analyses:** Cell lines are often used in research in place of primary cells. These *in vitro* analyses offer the advantages of low costs, ease of use, providing an unlimited supply of materials, and bypassing ethical concerns associated with the use of animal and human samples [101]. That said, the use of cell lines in cancer research has some drawbacks. A major drawback of in vitro models is their failure to capture the full reality of in vivo systems. For example, such models may not account for likely interactions between cells and other biological and biochemical processes. Cancer cell lines are often derived from a single subtype of tumor, which very often does not reflect the heterogeneity of tumors in patients. Furthermore, cancer cell lines are unlikely to reflect the tumor microenvironment, which is known to influence tumor progress and its response to therapy. Given that most reported investigations in the search for inhibitors to influence the expression of P-gp have been conducted on model cancer cell lines, it is reasonable to suggest that data generated by these analyses are less reliable and less informative than those conducted in vivo.

**- The multifactorial nature of drug resistance** is another factor to be considered as being partly responsible for the poor results in the search for specific inhibitors of P-gp. There is strong evidence that high expression of this protein remains one of the main reasons for the poor response to chemotherapy in many cancer types. That said, limiting the search for inhibitors to a single drug resistance mechanism is unlikely to restore chemotherapeutic sensitivity. As mentioned earlier, this protein is part of a superfamily containing at least 48 members. The existing literature shows that only three members have been studied in some detail. Currently we do not have sufficient structural and clinical information about most members of this superfamily. The scarcity of such information renders the answers to a number of questions regarding inhibitors of P-gp rather difficult to answer. For example, how common is the co-expression of multiple members within the same cell? How different is the molecular mechanism of substrate transport between one member and another? Will an inhibitor specifically designed to inhibit a given member restores chemotherapeutic sensitivity regardless of the expression of other members of the same family? These questions are difficult to answer without having more structural information together with a deeper insight into the biology of the various members of this family and their effectors The search for inhibitors of multiple transporters has been reported recently [120]. Tazemetostat (trade name Tazverik) is a novel epigenetic drug that has been recently approved for the therapy of advanced epithelioid sarcoma and follicular lymphoma. In their study, the authors investigated the inhibitory effects of Tazemetostat on selected ABC transporters (ABCB1, ABCC1, and ABCG2) and on the cytochrome P450 3A4 enzyme. The same study used accumulation and molecular docking studies to show that tazemetostat acts as a triple inhibitor of the three transporters, while a low level of interaction between the same reagent and the P450 3A4 enzyme was observed. Drug combination assays confirmed that tazemetostat is a multiple MDR modulator able to synergize with various conventional chemotherapeutics in vitro.

- **Ligand–protein interaction.** Over the last 40 years, chemical, biochemical, and biological studies have provided a wealth of information on the mechanisms of interaction between a limited number of ABC transporter proteins and various ligands. The complexity of such interaction renders such information insufficient to identify the precise mechanism(s) of such interactions. Identification of the basic physical and chemical characteristics, which mediate ligand–protein interaction are highly relevant for inhibitor(s) selection. In view of what we know about the capability of ABC transporters to bind to a wide range of structurally different substrates in the mass range 100–4000 Da, such a task is not easy to tackle. In more recent years, there have been promising steps towards this objective. For instance, there is an increased use of atomic structure resolution data of membrane proteins. Second, there is more frequent use of DHX-MS analyses to investigate the conformational states of various membrane proteins. Data generated by the above approaches are providing an insight into the mechanism of interaction between these proteins and structurally different substrates. Earlier studies reported multiple binding sites on individual transmembrane segments of P-gp that have the ability to simultaneously bind structurally different drugs or multiple molecules of the same structure [121,122]. These experimental data are considered a strong indication that the transmembrane segments (TMs) that contribute to the drug-binding pocket have a high degree of conformational mobility to allow drug molecules to form the required binding sites and to allow for different orientations of drug molecules within the binding pocket [104]. Furthermore, combinatorial chemistry [123] has been indicated as one of the tools to accelerate the screening of a high number of newly synthesized ligands and their interaction with membrane proteins. This technique permits the synthesis of structurally different chemical entities in extremely high numbers and in much shorter times compared to traditional methods of synthesis. This technique permits the generation of large chemical libraries, which can be tested against high numbers of targets in high-throughput screening.

**- Level of expression.** Accurate detection and quantification of ATP-binding cassette (ABC) transporters are highly relevant to the assessment of their role in chemoresistance. Such a parameter becomes more critical when expression is measured *in vivo*. Most studies conducted on cell lines indicate that the level of expression of one or more of these proteins varies from one tumor to another. The same literature shows that the detection and quantification of these levels have been mainly performed by reverse transcription–polymerase chain reaction (RT-PCR) for mRNA expression and by immunohistochemistry (IHC) for protein expression. Both methods have limitations, including normal tissue contamination of tumor tissue when total RNA methods are used. HIC low sensitivity and specificity, together with difficulties of quantification, render consistency and reproducibility in these analyses rather challenging. The high sensitivity and high specificity of mass spectrometry and its increased use in biological and biochemical analyses may contribute to the development of analytical platforms, which can be applied for accurate measurements of transporter expression, preferably in vivo. These types of analyses are further complicated by a wide range of responses to the various forms of cancer to chemo- and/or radiotherapies. Such a range of responses exhibits a spectrum which is difficult to rationalize by simply considering drug resistance.

**- The good and the bad.** ATP-binding cassette (ABC) transporters are expressed in both healthy and cancerous cells. Within the healthy cells, some of these proteins play a crucial physiological role in protecting tissues from toxic xenobiotics and endogenous metabolites, and also affect the uptake and distribution of many clinically important drugs. The same proteins form a major component of the blood–brain barrier and restrict the uptake of drugs from the intestine. In cancerous cells, on the other hand, the overexpression of these proteins has been strongly linked to MDR. Although MDR is multifactorial in origin, it is strongly associated with the overexpression of ATP-binding cassette (ABC) transporters. The above observations imply that the link between these proteins and MDR is mainly dependent on their identity, their level of expression, and on the environment where they are expressed. The same observations lead to an obvious question. Are we searching for inhibitors, which completely prevent the expression of these transporters, or are we searching for inhibitors which limit the expression to the level expressed in healthy cells? The answer to this question is contained within the existing literature, which indicates that the overexpression of such proteins is the main driver of this mechanism of resistance.

## 5. Conclusions

Mass spectrometry is becoming an increasingly important tool in the field of structural biology. In recent years, the role of this technique has been enhanced by more frequent use of ion mobility-MS, HDX-MS, and electron/photon-based ion activation methods. These techniques, together with X-ray crystallography and cryo-electron microscopy, are furnishing much-needed information on the structure of membrane proteins, including ABC transporters. Information provided by these techniques together with those acquired through advanced molecular dynamics simulation, molecular docking studies, and protein–ligand investigations will no doubt contribute to the ongoing scientific research to fill the gaps, which for over 40 years have hindered the identification of specific and effective clinical inhibitors of ABC transporters. The authors are convinced that the role of mass spectrometry will be further enhanced through a wider use of in vivo HDX-MS and more instrumental innovation to better exploit the potential of ion mobility–mass spectrometry and to accommodate different and more efficient ion activation methods.

Most of the works cited in this review agree that developing specific and effective inhibitors of P-glycoprotein and other transporter proteins is still challenging. The same works underline the following observations: (i) searching for inhibitors of P-gp without considering the influence of the other 47 members of the same family of proteins is unlikely to deliver the desired results; (ii) a limited number of these proteins have been examined by a host of techniques, including X-ray crystallography, cryo-electron microscopy, structural mass spectrometry-based proteomics, docking and protein–ligand studies, and other computational studies. To harvest the maximum benefits from the huge quantities of data generated by these techniques, such data sets have to be considered collectively and not singularly, as has been the case so far. (iii) More clinical trials based on in vivo samples and with phase III design are necessary, where patients are selected based on tumor expression of P-gp or other ABC transporters. Furthermore, to establish the impact of potential inhibitors in MDR, it is indispensable that such clinical trials select a group of patients where the overexpression of ABC transporters is considered as the main driver of one of the main mechanisms of multidrug resistance.

Currently there is a feeling of pessimism regarding the search for specific and effective inhibitors of (ABC) transporters. Such a pessimistic view is fueled by the setbacks of the last decades. The authors believe that recent advances in chemistry and biology and new datasets generated by proteomic, genetic, epigenetic, transcriptomic, and experimentally based computational modeling are likely to bring some optimism to the area of inhibitor discovery.

## Abbreviations

ATP	Adenosine triphosphate
MDR	Multidrug resistance
RND	Resistance-nodulation-division
SMR	Small multidrug resistance
MATE	Multi antimicrobial extrusion
MFS	Major facilitator superfamily
ATP	Adenosine triphosphate
MDR1. ABCB1.	Multidrug resistance protein1 (P-glycoprotein)
MDR	associated protein 1: MRP1; ABCC1
BCRP, ABCG2	Cancer resistance protein
MS	Mass spectrometry
ESI	Electrospray ionization
HDX-MS	Hydrogen/deuterium exchange mass spectrometry
TCA	Trichloroacetic acid
TCEP	Tris(2-carboxyethyl) phosphine
PTMs	Post-translational modifications
ECD	Electron capture dissociation
ETD	Electron transfer dissociation
IM-MS	Ion mobility–mass spectrometry
CCS	Collision cross-section
Q-TOF	Quadrupole/time-of-flight instrument
FT-ICR	Fourier transform ion cyclotron resonance
CID	Collision-induced dissociation
IRMPD	Infrared multiphoton dissociation
UVPD	Ultraviolet photodissociation
MALDI	Matrix-assisted laser desorption/ionization;
NBD	Nucleotide-binding domain;
TMD	Transmembrane domain;
FDA	Food and Drug Administration;
EMA	European Medicines Agency;
HCC	Hepatocellular carcinoma;
RT-PCR	Reverse transcription–polymerase chain reaction;
IHC	Immunohistochemistry;
NSCLC	Non-small cell lung cancer.
